# Joining of Dissimilar Alloys Ti-6Al-4V and Ti-6Al-2Sn-4Zr-2Mo-0.1Si Using Linear Friction Welding

**DOI:** 10.3390/ma13173664

**Published:** 2020-08-19

**Authors:** Sidharth Rajan, Priti Wanjara, Javad Gholipour, Abu Syed Kabir

**Affiliations:** 1 Mechanical and Aerospace Department, Carleton University, Ottawa, ON K1S 5B6, Canada; AbuSyedKabir@cunet.carleton.ca; 2 National Research Council Canada, Aerospace Research Center, Aerospace Manufacturing Technologies Center, 2107 chemin de la Polytechnique, Montréal, QC H3T 1J4, Canada; Priti.Wanjara@cnrc-nrc.gc.ca (P.W.); Javad.Gholipourbaradari@cnrc-nrc.gc.ca (J.G.)

**Keywords:** linear friction welding (LFW), solid-state joining, dissimilar alloy joints, post weld heat treatment (PWHT), stress relief annealing (SRA), titanium alloy, Ti-6Al-4V, Ti-6Al-2Sn-4Zr-2Mo-0.1Si, tensile properties, fractography

## Abstract

Dissimilar joints between Ti-6Al-4V (Ti-64) and Ti-6Al-2Sn-4Zr-2Mo-0.1Si (Ti-6242) were manufactured using linear friction welding. The weld quality, in terms of the microstructure and mechanical properties, was investigated after stress relief annealing (SRA) at 750 °C for 2 h and compared with the as-welded (AWed) results. The central weld zone (CWZ) microstructure in the AWed condition consisted of recrystallized prior-β grains with α’ martensite, which transformed into an acicular α+β structure after SRA. The hardness in the AWed condition was highest in the CWZ and decreased sharply through the thermomechanically affected zones (TMAZ) to the parent materials (PMs). After SRA, the hardness of the CWZ decreased, mainly due to tempering of the α’ martensite microstructure. Static tensile testing of the dissimilar welds in both the AWed and stress relief annealed (SRAed) conditions resulted in ductile fracture occurring exclusively in the Ti-6Al-4V side of the joint. The promising results on joining of Ti-64 to Ti-6242 provide valuable insight for tailoring performance of next-generation aero-engine products.

## 1. Introduction

Bladed integrated disks (Blisks)—manufactured by machining a single forged billet into a monolithic blade-disk assembly—were introduced in aero-engines as an advanced alternative [1] to mechanical fastening/joining of blades to disks via a fir-tree arrangement that faced service life limitations and failures due to fretting fatigue damage at the contact surfaces between the blade dovetail root and the disk slot [2]. Though the blisk technology demonstrated significant gains in weight reduction, aerodynamic performance, space reduction, product quality and service life, the monolithic manufacturing approach has encountered significant machining challenges [3], especially for blisks with high blade lengths in the low-pressure compressor stages. More importantly, however, has been the impossibility of mechanical performance tailoring in the monolithic blisk design through dissimilar alloy selection and microstructural modification for the specific operational conditions on the blades (exposed to high cycle fatigue and higher temperatures) relative to the disk (exposed to low cycle fatigue) [3]. This has led to avid research and development in the aerospace field over the past two decades on suitable joining technologies for titanium alloys [4,5,6,7,8,9], and in particular their solid-state assembly by linear friction welding (LFW) [10,11,12] that has now been qualified for aero-engine blisk manufacturing because the welds exhibit high integrity (free from defects such as porosity or inclusions), fine-grain “forged” microstructures in the central weld zone (CWZ) with limited thermomechanically affected zones (TMAZs) and heat affected zones (HAZs), as well as high joint efficiencies—ratio of the strength of the joint compared to the parent material (PM).

To date, published LFW research studies on titanium alloys have primarily focused on joining of similar titanium alloys and, most particularly, on the workhorse alpha-beta (α-β) Ti-6Al-4V (Ti-64) alloy with a bimodal microstructure, for which the influence of processing conditions has been inter-related to the metallurgical characteristics (microstructure, texture, etc.) and resulting mechanical properties (hardness, residual stresses, tensile, fatigue, etc.) in the as-welded (AWed) condition [13,14,15,16,17,18] and after post-weld heat treatment (PWHT) [19,20]. Other research studies on LFW of similar titanium alloys have included near-α titanium alloys, such as Ti-5.8Al-4Sn-3.5Zr-0.7Nb-0.5Mo-0.35Si-0.06C (IMI 834) [21] and Ti-6Al-2Sn-4Zr-2Mo-0.1Si (Ti-6242) [22], near-β alloys such as Ti-5Al-2Sn-2Zr-4Cr-4Mo (Ti-17) [23,24] and Ti-5Al-5Mo-5V-3Cr (Ti-5553) [25,26], as well as other α-β alloys such as Ti−4Al−4Mo−2Sn−0.5Si (IMI 550) [27] and Ti-6.5Al-3.5Mo-1.5Zr-0.3Si (TC11) [28]. Though the LFW technology is highly promising for joining two different materials together, reported research on weld joint assembly by LFW of dissimilar titanium alloys has been very limited in the open literature. In general, for welding dissimilar materials, the potential issues for attaining good metallurgical bonding and properties are exacerbated by, for instance, the differences in melting temperature and thermal expansion coefficients of the two materials, which may lead to higher thermal stresses and result in cracking in the transition zone between the materials. Previously, Baeslack et al. [29] studied LFW between Ti-6Al-2Sn-4Zr-2Mo-0.1Si (Ti-6242) and Ti-13.5Al-21.5Nb and reported that the weld interface was exposed to very high temperatures, which when combined with the occurrence of dynamic recrystallization and rapid cooling, resulted in the formation of a fine martensitic structure in the CWZ that had a very high hardness (~500 KHN). Corzo et al. [30] studied a hardness methodology to evaluate the yield strength of linear friction welded (LFWed) joints between Ti-64 and Ti-6246, as well as Ti-6242 and Ti-6246, but without comprehensive correlation of the mechanical properties to the microstructural characteristics. For LFW of Ti-64 to Ti-6246, Guo et al. [31] subsequently examined the microstructural transformations and microhardness evolution across the joint, both in the AWed and PWHTed conditions. They also reported the formation of fine martensite (α’) in the CWZ with concomitant peak hardness values, but there was no evaluation of the mechanical performance of the dissimilar joints.

Considering the limited reported research on LFW of dissimilar titanium alloys, the present study was undertaken to comprehensively evaluate the weldability of two industry-relevant titanium alloys: Ti-64 and Ti-6242. In particular, joining of Ti-64 and Ti-6242 is of interest for fan and compressor rotors operating at moderate temperatures and high loads. Industrial applications usually limit the use of the α-β Ti-64 alloy to about 350 °C [32], whilst the near-α Ti-6242 alloy—that is more oxidation and creep-resistant—can withstand maximum service temperatures of 510 °C [33]. As the comparable thermal and mechanical properties of Ti-64 and Ti-6242 provide good compatibility for joining using the LFW process, a dissimilar joint between these two titanium alloys is an opportunity to tailor product designs with locally higher temperature stability, as well as creep and oxidation resistance. Generally, the weldability of titanium alloys, including Ti-64 and Ti-6242, is limited by their high affinity for interstitial elements—oxygen, nitrogen and hydrogen. Thus, during conventional fusion welding techniques, shielding of the molten weld pool is key to preventing embrittlement of the fusion zone (from dissolution of interstitial elements) and/or formation of solidification defects (oxides, pores, etc.). Even with adequate shielding during fusion welding, coarsening of the microstructure occurs (i.e., large prior-β grain size and coarse α-β lamellae) in the weld zone [8] and HAZ [34] that limits performance under fatigue loading conditions. Thus, for titanium alloys, solid-state joining is especially propitious for preventing weld embrittlement and solidification defects, whilst enabling microstructural refinement in the CWZ.

To the knowledge of the authors, there have been no studies reported in the open literature on the joining of Ti-64 to Ti-6242 by LFW. In this work, the feasibility of manufacturing a dissimilar joint between Ti-64 and Ti-6242 was explored using the LFW process. Additionally, as the various studies to date on LFW of α+β titanium alloys have almost exclusively examined bimodal microstructures for the PM [11], the present study considered dissimilar microstructures for Ti-64 (equiaxed α with intergranular β) and Ti-6242 (primary-α globules in a transformed β matrix having a lamellar α morphology). The microstructural characteristics and microhardness evolution across the dissimilar Ti-64–Ti-6242 joints, as well as the tensile properties of the assembly were evaluated both in the AWed and stress relief annealed (SRAed) conditions.

## 2. Experimental Procedure

The materials selected in this study consisted of using Ti-64 (AMS 4911) and Ti-6242 (AMS 4919) alloys that were received in plate form with a thickness of 25 mm and a chemical composition as presented in Table 1. Chemical analysis of both alloys was performed using wavelength dispersive X-ray fluorescence spectrometry in accordance with ASTM E539 [35] for all elements except nitrogen, oxygen, hydrogen and carbon. Inert gas fusion was used to measure the hydrogen (ASTM E1447 [36]), nitrogen and oxygen (ASTM E1409 [37]) contents, while the carbon content was assessed by combustion analysis according to ASTM E1941 [38]. Typical properties of these alloy are shown in Table 2.

The coupons for LFW consisted of rectangular blocks—12.0 mm (W) by 24.5 mm (H) by 33.0 mm (L)—that were machined from the as-received Ti-64 and Ti-6242 rectangular plates with a tolerance of 0.02 mm. Just before placing the coupons in the welding fixture, the faying surfaces at the joint interface were lightly sanded using 320-grit sandpaper and then cleaned with ethanol.

**Figure 1:**
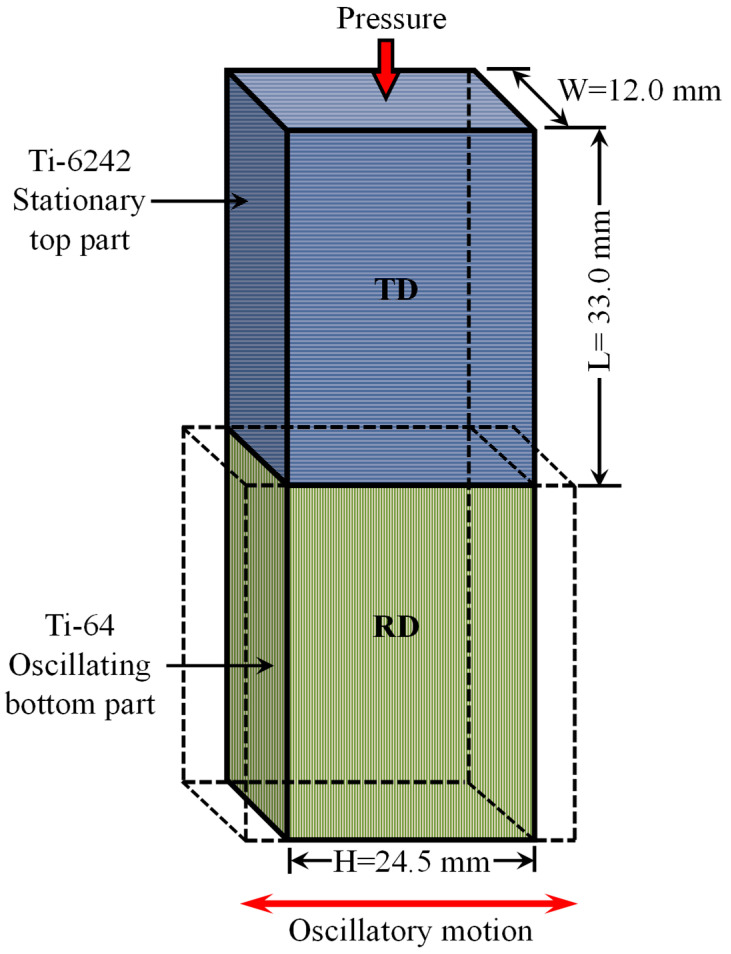
Schematic representation of length (L), width (W) and height (H) of the Ti-64 and Ti-6242 blocks with microstructures oriented in the rolling direction (RD) and transverse direction (TD), respectively.

LFW entails oscillating one part under an applied pressure against another stationary part, as illustrated in Figure 1. The pressurized oscillatory motion of the parts produces frictional heat and results in the plasticization of the interface material [39]. The plasticized material along with the interface contaminants, such as oxides, is then expelled in the form of flash toward the edges. Once the required axial shortening (burn-off) is achieved, a forge force is applied to consolidate the weld and effectively join the parts together.

The equipment used for LFW was an MTS LFW process development system (PDS) at the National Research Council Canada that comprised two hydraulic actuators: the in-plane actuator that oscillates the lower part (Ti-64) in the horizontal direction and the forge actuator that applies a downward load through the top stationary part (Ti-6242). The LFW experiments were conducted in air (without shielding gas protection) at an ambient temperature of 25 °C. More details about the technical specifications of the MTS LFW PDS system are provided in [39] and the process conditions used for LFW of the Ti-64 to the Ti-6242 are given in Table 3.

After LFW, selected dissimilar Ti-64-Ti-6242 welds were subjected to SRA at 750 °C for 2 h in a ceramic-tube vacuum-furnace. The vacuum pressure was maintained at 1.6 × 10^−2^ Pa to prevent oxidation of the samples. It is noteworthy that the temperature selected for SRA of the welds in the present research was based on the PWHTs conducted by Frankel et al. [19] for similar alloy linear friction welds, which indicated the lowest residual stresses using a stress relief annealing (SRA) temperature of 700 °C for Ti-64 and 800 °C for Ti-6242. Thus, in the present work, an intermediate temperature of 750 °C was chosen for the dissimilar Ti-64-Ti6242 welds.

**Figure 2:**
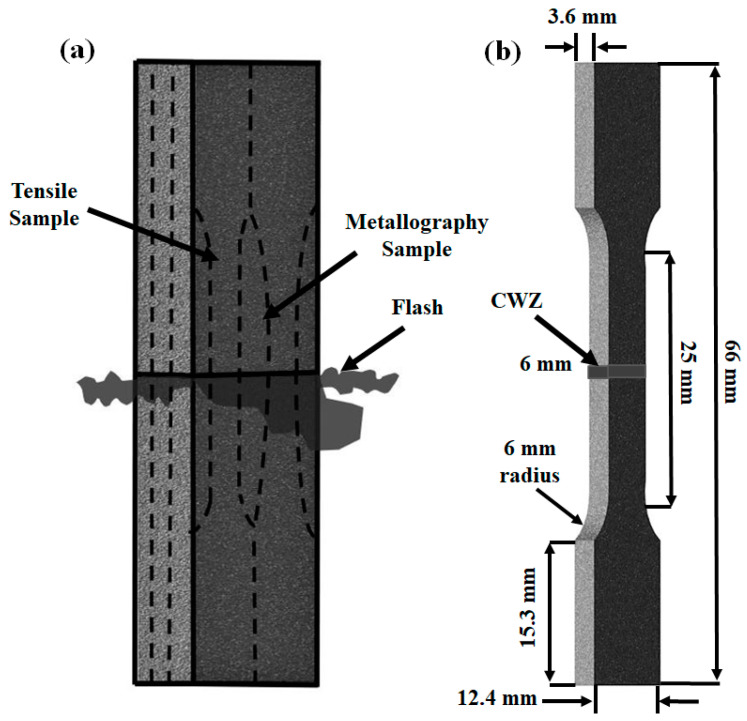
(**a**) Schematic representation showing the electrical discharge machining (EDM) plan for extracting the tensile and metallography samples from the welds and (**b**) geometry of the tensile samples.

Electrical discharge machining (EDM) (FANUC Robocut C400iB, Oshino, Japan) was used to extract metallographic and tensile samples from the AWed and SRAed Ti-6242 welds, as illustrated in Figure 2a. The metallographic samples extracted from the center of the Ti-64-Ti-6242 welds were prepared for microscopic examination by hot mounting in conductive resin (Struers ConduFast, Ballerup, Denmark) followed by automated grinding and polishing. Specifically, the samples were first ground planar using 280-grit SiC paper and water. Then the samples were ground on a Struers MD-Piano (1200 µm) pad (Ballerup, Denmark) with water as lubricant followed by MD-Largo (9 µm) with DP-blue lubricant. Final polishing was performed on a porous pad using a colloidal silica suspension (0.02 µm). After polishing, the samples were etched using Kroll’s reagent (100 mL of distilled water, 6 mL of nitric acid (HNO_3_), and 2 mL of hydrofluoric acid (HF)). The etching time varied between 6 and 12 s, depending on the region of interest (PM, HAZ, TMAZ, CWZ). Microstructural characterizations were performed using an Olympus GX71 optical microscope (OM) (Richmond Hill, Canada) and Tescan Vega-II XMU scanning electron microscope (SEM) (Warrendale, USA) at 20 keV. The volume fractions of the α and β phases were measured by thresholding image analysis. The circular intercept method of Heyn, Hilliard and Abrams (ASTM E112-13 [42]) was used to quantify the size of the primary α and transformed prior-β grains in the microstructure by (1) using a template of three concentric and equally spaced test circles with a total circumference of 500 mm on the micrograph, (2) counting the number of times each circular line segment intersected a grain boundary, and (3) calculating the ratio of intercepts to the circular line length. The use of circles as test lines was deemed appropriate to average the grain shape anisotropy of the rolled PM microstructures.

Microhardness testing across transverse weld sections (i.e., perpendicular to the oscillation direction) was performed on polished (mirror finished) surfaces of both the AWed and SRAed metallographic samples using a Struers DuraScan 80 hardness tester (Ballerup, Denmark) equipped with a motorized x-y stage and a built-in microprocessor for automated measurement of the hardness values. Hardness profiles were determined using an average of three measurements for each point with a load of 500 g and a dwell period of 15 s at an indent spacing of 0.2 mm. In addition, to better understand the hardness distribution, 3-dimensional (3D) hardness maps were generated over an area of 2.5 mm × 7.5 mm, covering both PM regions, as well as the HAZs, TMAZs and CWZ. It is noteworthy that the minimum test point separation distance for all measurements was at least three times the diagonal measurement of the indent to avoid any potential effect of strain fields from the neighboring indents.

Guided by the principles given in ASTM E8M-16a [43], standard sub-size tensile samples were extracted from the welds in the AWed and SRAed conditions and machined to the geometry shown in Figure 2b. The tensile samples were then mechanically tested at room temperature using a 250 kN MTS 810 testing frame integrated with a laser extensometer (Eden Prairie, USA). Tensile tests were conducted until rupture using displacement control at a rate of 0.125 mm/min, which corresponds to an average strain rate of 0.005 min^−1^. The tensile properties evaluated in this work included the yield strength (YS), ultimate tensile strength (UTS), and elongation (El) for each specimen. A minimum of three tensile samples in each condition (AWed and SRAed) were tested to calculate the average properties.

## 3. Results and Discussions

### 3.1. Macroscopic Examination

**Figure 3:**
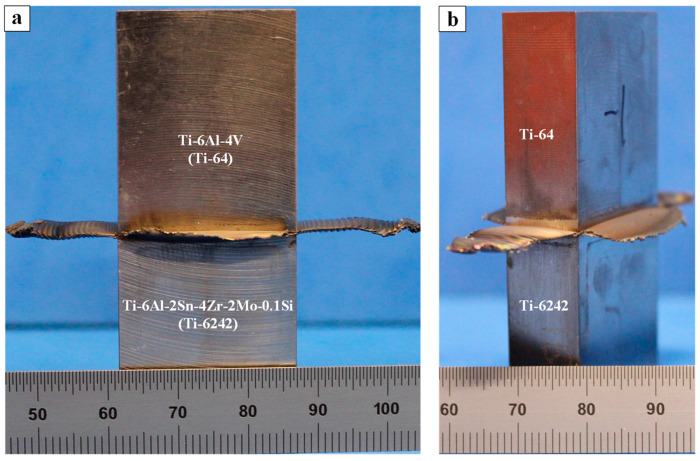
Linear friction welded specimen with flash extruded along the edges (**a**) front view (**b**) side view.

Visual inspection of the dissimilar titanium alloy welds revealed that the flash layer extruded practically equally from both the Ti-64 and Ti-6242 materials, as shown in Figure 3a,b. Specifically, along all four sides of the joint interface, appreciable flash was observed and the single joined flash layer on each side comprised of plastically deformed Ti-64 and Ti-6242 materials, as a combined effect of the applied frictional force and the reciprocating motion. Flash generated along the reciprocating direction was found to be longer than along the specimen width. Additionally, the flash layers along the width exhibited surface ripples or ridges (Figure 3a) as a result of the oscillatory motion during the LFW process that rendered stepwise extrusion of the material from the interface. By contrast, the flash layers along the height exhibited little to no evidence of ridges (Figure 3b).

Previously, Baeslack et al. [29] reported a uniform flash for dissimilar joints between Ti-6242 and Ti-13.5Al-21.5Nb with preferential extrusion of the lower strength (at elevated temperatures) alloy, namely Ti-6242. By contrast, Ma et al. [44] reported that the joint between Ti-64 and Ti-6.5Al-3.5Mo-1.5Zr-0.3Si (TC11) had appreciable flash on all sides with a resemblance to similar alloy linear friction welds in TC11 [28] and Ti-64 [39] alloys. These similar alloy welds [28,39] exhibited single joined flash layers on all four sides of the joint and the flash layer along the specimen height showed little evidence of ridges, while that along the specimen width had a series of surface ripples due to the oscillation in the direction of the specimen height. Thus, the uniformity and occurrence of single joined flash layers during LFW of dissimilar titanium alloy welds appear to be dependent on strength differences between the two materials at elevated temperatures, comparable to other dissimilar alloy joints [45,46,47,48,49]. Considering that temperatures during LFW of titanium alloys are typically above the β transus, both Ti-64 and Ti-6242 in the β phase field have similar compressive strengths of about 60 MPa at 1000 °C [49,50]. These reported findings thus corroborate the results in the present work on the flash morphology characteristics in the dissimilar Ti-64-Ti-6242 linear friction welds.

### 3.2. Microscopic Examination

**Figure 4:**
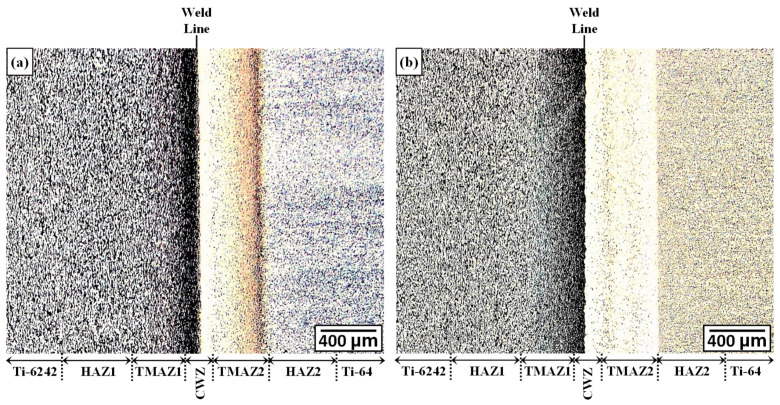
Optical Microscope (OM) images showing the different regions across the dissimilar Ti-64-Ti-6242 welds: (**a**) as-welded (AWed) and (**b**) stress relief annealed (SRAed) conditions.

Microscopic evaluation of the dissimilar Ti-64-Ti-6242 welds was undertaken for both the AWed and SRAed conditions. At low magnification, a weld line separating the Ti-6242 and Ti-64 sides could be clearly observed from the OM images given in Figure 4a,b. On the basis of nomenclature defined in previous studies [10,11], six different microstructural regions across the dissimilar Ti-64-Ti-6242 welds were identified, namely the Ti-6242 PM, Ti-64 PM, TMAZ1 and HAZ1 on the Ti-6242 side, TMAZ2 and HAZ2 on the Ti-64 side and the CWZ, as indicated in Figure 4. The boundaries of these regions were based on differentiating microstructural features, such as grain elongation/deformation in the TMAZ, as well as phase transformations and recrystallization in the CWZ. Additionally, from microscopic examination it was difficult to discern the presence of the HAZ between the PM and TMAZ on each side of the dissimilar joints for both the AWed and SRAed conditions; thus the boundaries delineated for HAZ1 and HAZ2 on the Ti-6246 and Ti-64 sides, respectively, were distinguished from the hardness changes across the AWed and SRAed dissimilar joints, as discussed later in the next section. It is interesting to note the comparable size of the CWZ and TMAZ on the Ti-64 and Ti-6242 sides of the dissimilar joints in the AWed condition. From microscopic examination, the plastically deformed zone (PDZ)—covering the combined thickness of the CWZ and TMAZ—was ~500 µm for both the Ti-64 and Ti-6242 sides. This may be attributed to the similar high temperature strength of Ti-6242 and Ti-64 [50,51], as well as their comparable temperature-dependent thermal conductivity behavior and values [52,53,54,55]. Previously in the work of Guo et al. [31] on LFW of Ti-64 to Ti-6246, the size of the PDZ was also similar, at about 1.5 mm on either side of the weld line. By contrast, Boyat et al. [56] recently indicated that dissimilar joints between Ti17 and Ti-6242 manufactured by LFW have different PDZ sizes of approximately 1 mm and 2.5 mm in the Ti-6242 and Ti17 sides, respectively. Wen et al. [57] LFWed Ti-64 to TC11 and also reported different PDZ sizes of ~1.1 mm on the Ti-64 side and ~1.7 mm on the TC11 side; they attributed this occurrence to the higher thermal conductivity of the TC11 relative to the Ti-64. Thus, joints between dissimilar, but mutually soluble, titanium alloys have PDZs with affected microstructures on either side of the weld line, and their relative size is dependent on the thermal conductivity and strength of the respective alloys at the elevated temperatures transpiring during the LFW process. This finding is of course in contrast to joints between dissimilar but incompatible alloys where the size of the PDZ on either side of the weld line is primarily dependent on the high temperature strength of each alloy [44,45,46,47,48].

#### 3.2.1. Characteristics of the PMs

**Figure 5:**
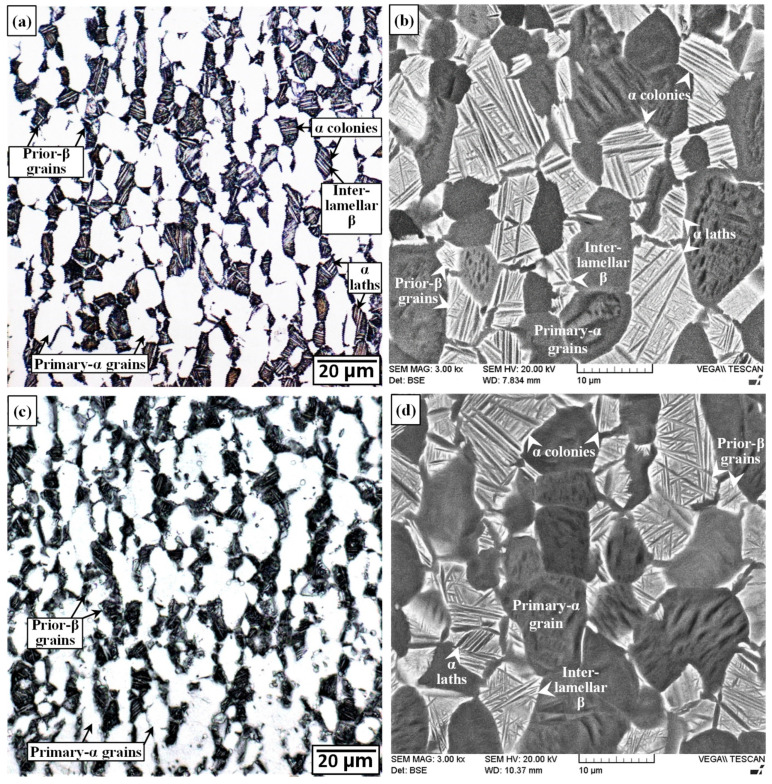
Microstructure of Ti-6242 PM (**a**,**b**) as-received (OM and SEM) and (**c**,**d**) SRAed (OM and SEM).

The as-received microstructure of the Ti-6242 alloy is shown in the representative OM and SEM images given in Figure 5a,b. The Ti-6242 alloy exhibited a bimodal microstructure consisting of elongated primary-α and transformed prior-β grains with an average size of about 17 µm and 18 µm, respectively. The measured volume fraction of the primary-α was about 52%. As demarcated in Figure 5a,b, the globular primary-α grains are the bright and dark regions, respectively, in the OM and backscattered electron (BSE) SEM images. By contrast, the transformed prior-β grains appear darker than the primary-α grains in the OM image (Figure 5a), but are brighter than primary-α grains in the BSE SEM image (Figure 5b). This is because in the BSE SEM images, the darker regions are the α phase due to the locally higher content of aluminum (lower atomic number) relative to β phase that has a higher local content of vanadium (higher atomic number). Within the transformed prior-β grains, the secondary α microstructure was predominantly Widmanstätten, consisting of randomly oriented α-β lamellae. Within some transformed prior-β grains, colony morphologies were also observed that consisted of multiple colonies of sandwiched α-β phase laths. In the BSE SEM images, these α and retained β phases are discernable, respectively, as dark and bright laths, which are demarcated in Figure 5b.

As illustrated by the representative OM and BSE SEM images given in Figure 5c,d, the microstructural characteristics of the Ti-6242 alloy after SRA at 750 °C for 2 h was quite similar to the as-received condition, consisting of a bimodal microstructure of elongated primary-α and transformed prior-β grains within which the secondary α morphology was predominantly Widmanstätten with some colonies also present. According to the thermodynamics of phase transformations in the Ti-6242 alloy, the volume fraction of the primary-α globules in the microstructure is temperature dependent. Specifically, heat treatment of the as-received bimodal microstructure can lead to decreases in the volume fraction of the globular primary-α phase with increasing temperature due to the α + β ⟶ β transformation. For a Ti-6242 alloy with a bimodal microstructure, Semiatin et al. [58] studied the α-β phase transformations with increasing temperature and determined that the amount of the globular primary-α phase decreased by 5%—from 60% in the as-received microstructure to 55%—after heat treatment at ~900 °C for 2 h. Their work indicated more noticeable decreases in the amount of globular α near the β transus temperature (990 °C); as such, holding at ~980 °C for 2 h halved the amount of globular α relative to the as-received microstructure [58]. This reasonably supports the observations and findings in the present work that indicated no discernible changes in the bimodal microstructure of the Ti-6242 alloy after SRA at 750 °C for 2 h.

**Figure 6:**
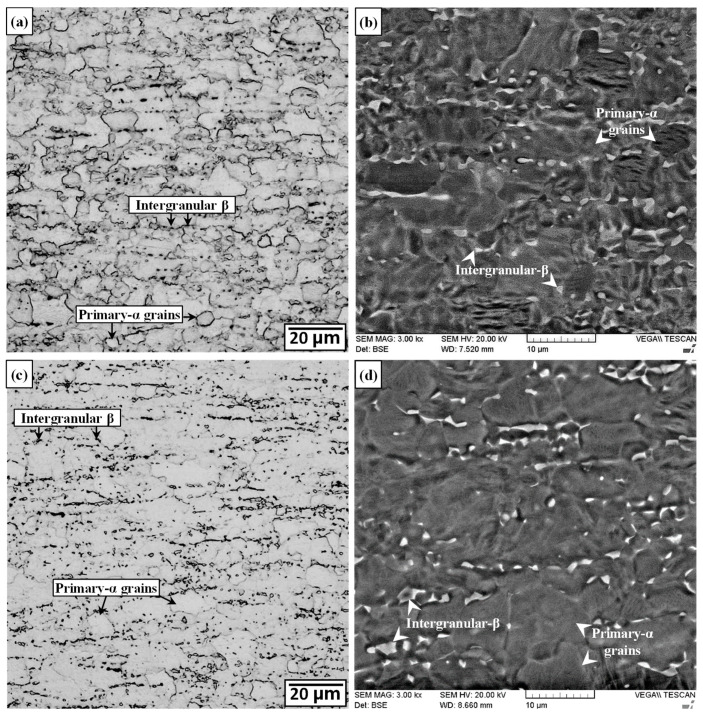
Microstructure of Ti-64 PM (**a**,**b**) as-received (OM and SEM) (**c**,**d**) SRAed (OM and SEM).

As shown in Figure 6a,b, the as-received microstructure of the Ti-64 alloy consisted of slightly elongated primary-α grains that were oriented parallel to the rolling direction and had an average size of roughly 12 µm. At the “triple points/junctions” and/or boundaries of these α grains, a small fraction of intergranular β was also observed. The measured volume fraction of the α and β phases was 94% and 6%, respectively. The formation of these primary-α grains in Ti-64 is derived from the mill annealing process that involves hot working in α+β phase field to nucleate (at primary-/prior-β grain boundaries) new, recrystallized α nuclei, which then grow into grains by “consuming” the high stored (deformation or strain) energy during slow cooling or subsequent annealing. In the present work, the elongated morphology of primary-α grains suggests that the as-received Ti-64 plate was lightly worked as heavily deformed material typically recrystallizes fully and has equiaxed primary-α grains.

After SRA at 750 °C for 2 h, both the OM and BSE SEM images of the Ti-64 alloy indicated minor changes in the fraction (5%) of intergranular β, as illustrated in Figure 6c,d, respectively. Similar to the Ti-6242 alloy, phase transformations in Ti-64 are also temperature dependent and, with increasing temperature, the volume fraction of the primary-α phase would decrease due to the α + β ⟶ β transformation. Yu et al. [59] studied this transformation for an equiaxed microstructure of primary-α grains with intergranular β and determined that at low heating rates (0.1 °C·s^−1^), noticeable transformation of the α phase to β begins only at 873 °C. Pederson et al. [60] investigated phase transitions in Ti-64 with a bimodal microstructure using high temperature X-ray diffractometry and observed no change in the α/β fractions during isothermal holding at 610 °C, but the percentage of β increased from ~4% at room temperature to 12% at 710 °C during isothermal holding for 2 h. Nonetheless reverse transformation on cooling to ambient temperatures, decreased the β fraction from 12% at 710 °C to ~3% at room temperature—that is, slightly lower than the amount in the as-received microstructure [60]. For higher heat treatment temperatures, the β fraction increased considerably and, during isothermal holding at 800 °C, the microstructure contained 40% β after 2 h, but upon reverse transformation to room temperature, the amount of β was only ~6% (i.e., roughly 2% higher than that in the as-received microstructure) [60]. These previous findings corroborate well with the observations in the present work that indicated only a minor change in the characteristics of the intergranular β in the room temperature microstructure of the SRAed Ti-64 material relative to the as-received alloy.

#### 3.2.2. Characteristics of the CWZ

**Figure 7:**
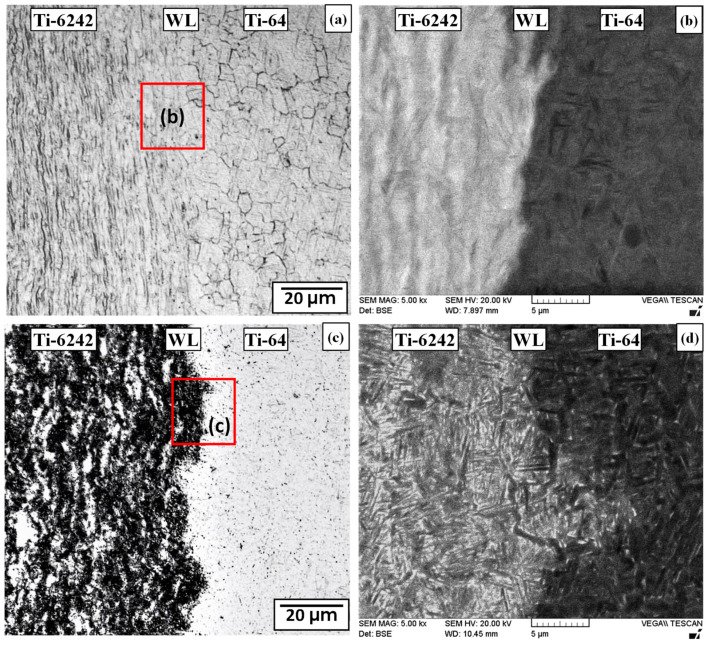
Microstructures in central weld zone (CWZ) on either side of the weld line (WL) separating the Ti-6242 and Ti-64 sides (**a**,**b**) AWed (OM and SEM) (**c**,**d**) SRAed (OM and SEM).

Examination of the CWZ distinctly revealed a weld line at the initial friction welding interface between the Ti-64 and Ti-6242 alloys (Figure 4). As illustrated by both the OM and SEM images given in Figure 7, the weld line separating the two sides of joint was wavy in appearance. The occurrence of this stark microstructural contrast at the weld line is indicative of a lack of any long-range ordering on diffusion across the initial interface, which may be attributed to the fact that the interface experiences temperatures above the β-transus for a very short duration that limits atomic diffusion and element partitioning during LFW. The observation of a weld line in the present work is consistent with previous dissimilar welding studies on Ti-64 and Ti-6246 conducted by Corzo et al. [30] and Guo et al. [31], as well as Ti-64 and TC11 performed by Wen et al. [57] and Ma et al. [44].

Examination of the CWZ from edge to edge showed no signs of any defects, such as cracks, oxides, pores, or micro-pores, indicating that the dissimilar welds between Ti-64 and Ti-6242, produced under the set of process parameters given in Table 3, are integral. By contrast, Boyat et al. [56] recently reported the presence of spherical micro-pores roughly <1 µm in diameter at the weld line of dissimilar welds between Ti-64 and Ti-17 that were produced under very similar conditions of a pressure of 90 MPa, frequency of 50 Hz, amplitude of 2 mm and axial shortening of 5 mm. The incomplete mechanical bonding/closure of interfacial voids in their work indicates that their specific power input during LFW was insufficient [13] for an integral weld between Ti-64 and Ti-17, possibly stemming from the larger cross-sectional area and size of their workpieces [56].

Relative to the microstructural characteristics of the Ti-64 and Ti-6242 PMs, the CWZ on both sides of the weld line exhibited entirely different microstructures consisting of fine acicular α features within refined prior-β grain boundaries, as indicated in Figure 7. For instance, in the AWed condition, the OM image (Figure 7a) reveals the presence of fine equiaxed prior-β grains, with an average grain size of 4.1 µm, just adjacent to the weld line on the Ti-64 side of the CWZ. For the Ti-64 alloy, this microstructural evolution can be reasoned from the progressive transformation of α with increasing temperature (α + β ⟶ β). Specifically, frictional heating during LFW rapidly increased the temperature and, in the CWZ, where the highest temperature occurred, the predominantly α grain structure of the PM transformed completely to the β phase. Considering the nominal β-transus temperature for Ti-64 (Table 1), the transformed β microstructure in the CWZ indicates that the local temperatures surpassed 980 °C, which is in agreement with previously reported experimental [14,44] and finite element modelling research [61,62,63,64,65] on LFW of Ti-64. Relative to the Ti-64 PM microstructure, the equiaxed and refined morphology of the transformed β grains in the CWZ is also inherent to the LFW process that produces thermomechanical conditions of large strains at high strain rates in the single-phase β-field that greatly exceed the critical values necessary to produce dynamic recrystallization in the alloy, as reported in [45,63,64,66,67]. Additionally, the martensitic (α’) morphology (Figure 7b) of the transformed β microstructure within the prior-β grains is a result of rapid cooling after LFW, which for the Ti-64 alloy transpires as a displacive (diffusionless) transformation at rates greater than 410 °C·s^−1^, according to Ahmed and Rack [68].

On the Ti-6242 side, the AWed microstructure just adjacent to the weld line in the CWZ also consisted of recrystallized prior-β grains (Figure 7a) that had an equiaxed morphology with a refined grain size (roughly 3.5 µm). Within these recrystallized prior-β grains the microstructure was martensitic in appearance with the α’ laths arranged as brick wall-like structures (Figure 7b). Similar to the transformations on the Ti-64 side, the CWZ on the Ti-6242 side also experienced complete transformation of the α phase to β, which then dynamically recrystallized under the thermomechanical conditions experienced during LFW—that consisted of super-transus temperatures with high strains and strain rates—and, subsequently on rapid cooling, transformed to α’. These observations are consistent with the findings from previous studies on LFW of similar Ti-6242 joints that reported a CWZ showing evidence of complete phase transformation from α to β [69], dynamic recrystallization of the prior-β phase [69], and brick wall-like arrangement of α’ laths formed within the transformed prior-β grains [19]. Moreover, previous studies on LFW of dissimilar titanium alloys [30,31] reported similar microstructural transformations at the weld interface in the CWZ.

After SRA for 2 h at 750 °C, the microstructure in the CWZ was noticeably different (Figure 7c,d) relative to the AWed condition. Though the size of the prior-β recrystallized grains after SRA remained comparable to the AWed condition, the α’ structure transformed during the tempering PWHT and, on both the Ti-64 and Ti-6242 sides of the CWZ, thickening of the laths was observed, as visible in the BSE SEM image shown in Figure 7d. In Ti-64, the decomposition of α’ to α and β phases during isothermal heat treatments has been reported to occur by a nucleation and growth process that is controlled by element diffusion and partitioning of aluminum and oxygen that accumulate in the α’/α phase(s) and the vanadium and iron that segregate and concentrate in the β phase [70]. Motyka et al. [71] studied heat treatment of undeformed α’ in Ti-64 and reported that the decomposition of α’ at 750 °C after 2 h results in α-lath thickening, which may be attributed to the heterogeneous precipitation of fine β phase platelets that occurs predominantly along α’ basal planes or lath boundaries and occasionally within the laths [72,73]. In addition, LFW of similar alloy joints in Ti-64 by Karadge et al. [20] and in Ti-6242 by Ballat-Durand et al. [74] reported α’ decomposition to acicular α and β phases after PWHT. These reported findings corroborate well with the microstructural observations in the present study that indicate thickening of the α-laths due to α’ decomposition in the CWZ on both the Ti-64 and Ti-6242 sides of the dissimilar alloy joint in the SRA condition.

#### 3.2.3. Characteristics of the TMAZs

**Figure 8:**
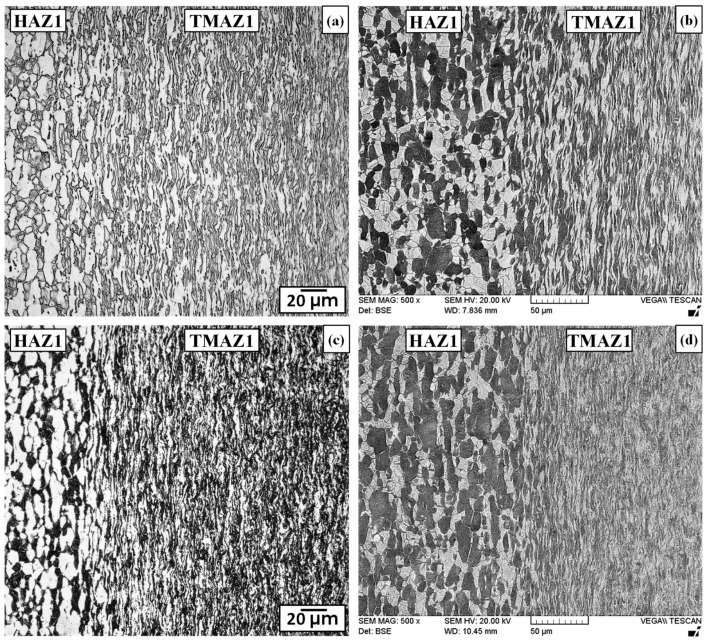
Microstructures in thermomechanically affected zone (TMAZ)1 between the CWZ and heat affected zone (HAZ)1 next to the Ti-6242 PM (**a**,**b**) AWed (OM and SEM) (**c**,**d**) SRAed (OM and SEM).

**Figure 9:**
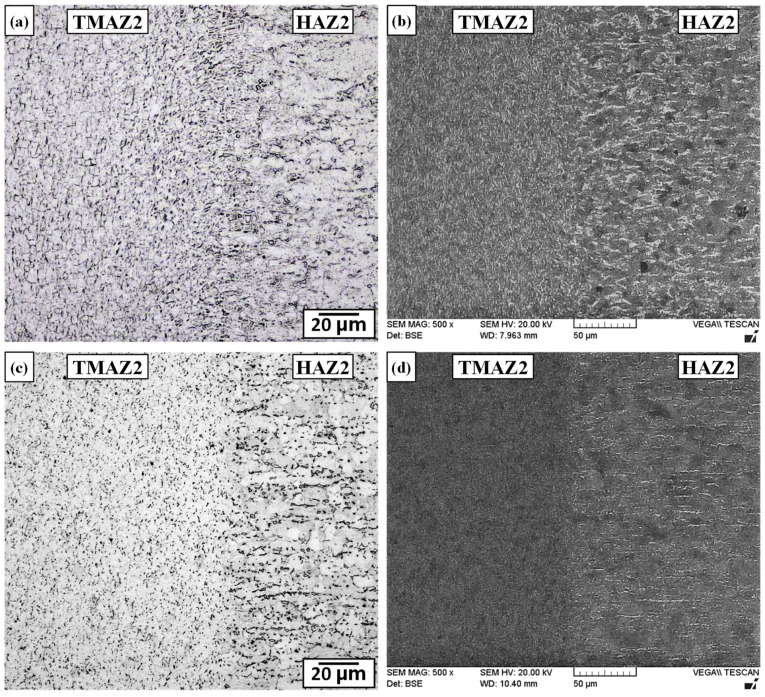
Microstructures in TMAZ2 between the CWZ and HAZ2 next to the Ti-64 PM (**a**,**b**) AWed (OM and SEM) (**c**,**d**) SRAed (OM and SEM).

Starting at about 100 µm from the weld line on both sides of the dissimilar alloy joint, a narrow TMAZ formed between the CWZ and HAZ on the Ti-6242 and Ti-64 sides, which was designated as TMAZ1 and TMAZ2, respectively. Both TMAZ1 and TMAZ2 experienced a continuous range of sub-transus temperatures and deformation conditions that decreased progressively from the CWZ to the unaffected PM. Thus, relative to the CWZ, the dynamic recrystallization kinetics were lower in TMAZ1 and TMAZ2, which resulted in high deformation and elongation of the grain structure in the oscillation direction. For instance, the AWed microstructure of TMAZ1 in Figure 8a,b shows deformed/distorted primary-α and transformed β grains that become increasingly fibrous and fragmented in appearance from HAZ1 to the CWZ on the Ti-6242 side. Alongside this clear evidence of severe plastic deformation, the gradual dissolution of the primary-α grains with the concomitant increase in the transformed β fraction from HAZ1 to the CWZ also suggest that the temperatures in TMAZ1 remained below the β transus. Similarly, the AWed microstructure in TMAZ2 showed grains reoriented along the oscillation direction, as well as a gradual dissolution of the primary-α grains and, concurrently, a progressive transformation of the intergranular β phase into transformed β grains, as illustrated in Figure 9a,b. Close to the CWZ boundary, the microstructure consisted predominately of dynamically recrystallized prior-β grains with some small fragmented and elongated α grains that point to the sub-transus temperature conditions in TMAZ2. The gradual transformation of the microstructural phases and grain structure characteristics observed in the present study agree with those reported previously on similar welds of Ti-64 [14,39] and Ti-6242 [74].

Differentiating microstructural changes in the TMAZ1 and TMAZ2 after SRA was not possible using an OM, as indicated by Figure 8c and Figure 9c, respectively. The effect of SRA on the microstructures in TMAZ1 and TMAZ2 was most apparent through BSE imaging with a SEM, as shown in Figure 8d and Figure 9d, respectively. As mentioned above, in BSE SEM imaging, regions with a higher vanadium content (higher atomic number) appear brighter and correspond to the β phase; by contrast, darker regions are higher in aluminum (lower atomic number) content and correspond to the α phase. Thus, directly comparing the bright phase regions in the AWed and SRAed microstructures point to a higher fraction of vanadium-rich β regions in TMAZ1 and especially TMAZ2 on cooling after LFW. Considering the temperature dependence of element partitioning effects in α+β alloys, Huang et al. [75] reported that while the concentration of stabilizers in the primary-α phase remain nearly constant, the transformed β phase experiences compositional variations, especially in relation to vanadium enrichment, which occurs proportionally to the volume fraction of the β phase existing at the process temperatures before cooling. Oh et al. [76] reported that vanadium enrichment has a stabilizing effect on the β phase and, at a vanadium content <4.3%, the β phase readily transforms to α’ on rapid cooling, but at higher levels, the β phase is retained. Considering the sub-transus temperatures in TMAZ1 and TMAZ2, the volume fraction of the β phase formed during LFW decreases with temperature, which then causes progressively higher vanadium enrichment (from the CWZ to the HAZ) and a higher likelihood of metastable β retention at room temperature. In this regard, the smaller fraction of β in TMAZ2 (equiaxed α with intergranular β microstructure) relative to TMAZ1 (bimodal microstructure) would also result in higher vanadium enrichment and metastable β retention in TMAZ2. Subsequently, diffusion of vanadium during SRA leads to homogenization and equilibration of elements in the microstructure, such that on slow cooling to room temperature, the metastable β phase transforms readily to equilibrium levels of α-β phases. The retention of metastable β in the TMAZ of similar titanium alloy welds has been reported previously by Karadge et al. [20] for Ti-64 and Frankel et al. [19] for Ti-6242, which both had starting (PM) microstructures that were bimodal. Recently, Ballat-Durand et al. [74] studied LFW of bimodal Ti-6242 and separated the TMAZ into two regions—near TMAZ and far TMAZ relative to the weld line—based on the different microstructural constituents they observed with X-ray diffraction that indicated the retention of β in the far TMAZ. After PWHT, Frankel et al. [19] reported that the metastable β formed in the TMAZ of similar titanium alloy welds (Ti-64 and Ti-6242) transformed to an acicular α-β microstructure. These previously reported findings support the observations in the present study of retained β formation in TMAZ1 and TMAZ2 of the dissimilar joint between Ti-64 and Ti-6242, and its subsequent transformation to α-β constituents during SRA.

#### 3.2.4. Characteristics of the HAZs

Similar to conventional fusion welds, the HAZ generated during LFW is defined as a region that has been affected only by heat from the process. Hence, the material in HAZ1 and HAZ2 experiences temperature gradients from the LFW process without mechanical deformation effects. The AWed and SRAed microstructures are shown in Figure 8 for HAZ1 and Figure 9 for HAZ2. Though microstructural changes were not clearly evident optically, BSE imaging with a SEM showed higher fractions of bright retained β regions in the AWed microstructures of HAZ1 and HAZ2 relative to the SRAed condition. In addition, without the convoluting effects of plastic deformation (as in the case of the TMAZ), the increased fraction of metastable β is readily noticeable in the AWed microstructure of both HAZ1 and HAZ2. SRA effectively transformed the metastable β, and the (equilibrium) microstructure in HAZ1 and HAZ2 had a higher fraction of α relative to the AWed state. Reasoning for the retention of metastable β in HAZ1 and HAZ2 on cooling after LFW and its subsequent transformation during SRA is similar to that explained above for TMAZ1 and TMAZ2.

## 4. Hardness

**Figure 10:**
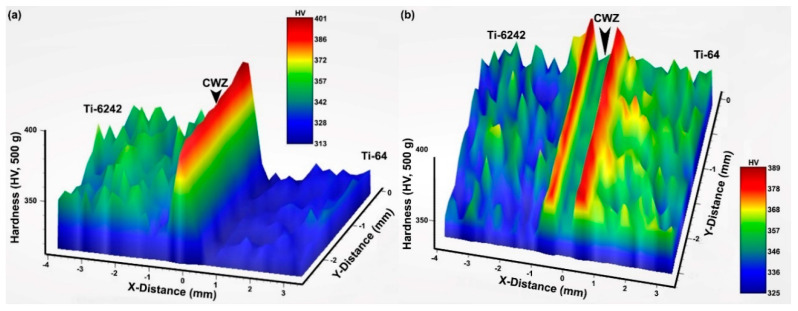
3D microhardness maps across the dissimilar linear friction welds between Ti-64 and Ti-6242 in (**a**) AWed and (**b**) SRAed conditions.

For the AWed and SRAed conditions, 3D hardness maps were generated over an area that adequately covered the different regions of the linear friction weld from the Ti-6242 PM through the CWZ and to the Ti-64 PM. As shown in Figure 10, these 3D plots provide an overview of the Vickers hardness evolution across the transverse cross-section of the dissimilar joint and depict the effect of non-homogeneities in the PM microstructure (hard α and soft β phases), as manifested by the hardness variations in the Ti-6242 and Ti-64 areas. In the AWed condition, a peak hardness of 401 HV0.5 occurred in the CWZ at the weld line (distance of x = 0) separating the two alloys. Additionally, just adjacent to the PDZ, softening in the Ti-6242 and Ti-64 occurred with the presence of hardness minima of 330 HV0.5 and 313 HV0.5, respectively. SRA had the effect of significantly reducing the hardness in the CWZ to a value of 350 HV0.5 at the weld line, as well as recovering the softened regions next to the PDZ on both the Ti-6242 and Ti-64 sides of the joint. Furthermore, while softening of the Ti-6242 PM was not significant (5 ± 8 HV0.5), the Ti-64 PM hardened by 30 ± 5 HV0.5 after SRA at 750 °C for 2 h.

**Figure 11:**
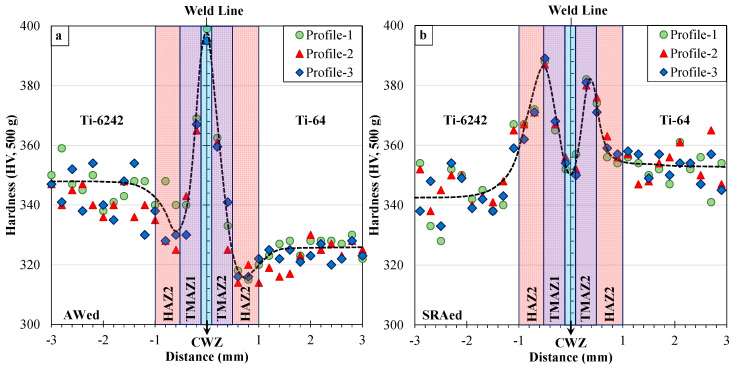
Microhardness profiles across the weld line in dissimilar linear friction welds between Ti-64 and Ti-6242 in (**a**) AWed and (**b**) SRAed conditions.

Figure 11 shows the hardness evolution across the dissimilar alloy joint using three profiles measured in the middle (profile 1) and two edge (profile 2 and 3) areas of the weld in the AWed and SRAed conditions. Overall, these hardness profiles corroborate well with the underlying hardness trends observed from the 3D hardness maps of the AWed and SRAed welds. However, use of the linear profiles facilitated linking of the hardness evolution to the seven different regions in the dissimilar alloy joints—namely Ti-6242 PM, HAZ1, TMAZ1, CMZ, TMAZ2, HAZ2 and Ti-64 PM (Figure 4). In general, the hardness variations across the dissimilar alloy joints aligned suitably with the microstructure changes observed. For instance, the occurrence of hardness maxima (398 ± 3 HV0.5) at the weld line in the CWZ is attributed to the α’ microstructure of the refined (recrystallized) transformed prior-β grains. SRA had the effect of tempering/decomposing the α’ to α and β phases in the CWZ, which reduced the average hardness at the weld line to 367 ± 2 HV0.5 (i.e., 8% reduction). Relative to the CWZ, the average AWed hardness in TMAZ1 (370 ± 5 HV0.5) on the Ti-6242 side and TMAZ2 (360 ± 3 HV0.5) on the Ti-64 side were lower due to the locally lower kinetics for dynamic recrystallization that limited the plastic deformation and resulted in a microstructure consisting predominantly of fragmented remnant primary-α and transformed β grains with some α’ within recrystallized prior-β grains. On cooling, evidence of metastable β retention in TMAZ1 and TMAZ2 also contributed to lowering the hardness, especially close to HAZ boundary. During SRA, the α’ and retained β phases decomposed or transformed to α and β phases, and contributed to, respectively, decreasing (α’ ⟶ α + β) and increasing (β_retained_ ⟶ α + β) the hardness. Under these opposing effects, the average hardness in TMAZ1 (370 ± 15 HV0.5) and TMAZ2 (369 ± 12 HV0.5) after SRA remained comparable to the AWed values, despite noticeable changes in the microstructure. The occurrence of hardness minima in HAZ1 (333 ± 6 HV0.5) on the Ti-6242 side and HAZ2 (315 ± 2 HV0.5) on the Ti-64 side of the AWed joints is associated with the retention of metastable β (soft) in the room temperature microstructure that lowers the hardness relative to that of the as-received Ti-6242 (345 ± 8 HV0.5) and Ti-64 (326 ± 4 HV0.5) PMs. After SRA, the average hardness in HAZ1 (368 ± 4 HV0.5) and HAZ2 (357 ± 3 HV0.5) recovered to values just slightly higher than that of the Ti-6242 (342 ± 9 HV0.5) and Ti-64 (352 ± 5 HV0.5) PMs (in the SRA condition), respectively, due to the transformation of the metastable β phase that increased the α fraction and lowered the β fraction in the resulting equilibrated microstructure. This same reasoning (transformation of metastable β) also accounts for the minor hardening of the Ti-64 PM after SRA at 750 °C as the microstructure indicated a minor decrease in the intergranular β phase fraction relative to as-received mill-annealed condition. It is worth mentioning that at 750 °C—the temperature used in the present study for SRA—age hardening of Ti-64 is not possible as its solvus temperature is 550 °C for precipitation of Ti_3_Al. In the case of the Ti-6242 PM, there were no significant changes in the microhardness and the bimodal microstructure between the as-received and SRA conditions, which is reasonable considering its transformation characteristics (as discussed above) and solvus temperature (650 °C) for Ti_3_Al precipitation.

In general, previous studies on LFW of similar titanium alloys have also indicated hardness peaks in the CWZ and reported hardness increases of 18–40% in the AWed condition relative to the as-received PM (Table 4). Of greater controversy has been the TMAZ and/or HAZ hardness values, which were reported to be in-between those of the CWZ and PM [15,39,51,77,78,79] or lower than the PM [16,17,39,53,79]. Grujicic et al. [62] attributed the lower hardness in the TMAZ/HAZ to grain coarsening. By contrast, Lütjering and Williams [80] indicated that superior hardness in the TMAZ/HAZ required increases in cooling rate to prevent growth of grain boundary and α phase laths in the TMAZ/HAZ. On the other hand, Stinville et al. [17] reported a hardness drop in the HAZ, but their detailed examination of the α/α’ microstructure using electron backscatter diffraction showed no differences relative to the PM phase constituents. Romero et al. [16] and Wanjara and Jahazi [39] described the influence of LFW process parameters (e.g., amplitude and frequency) on lowering the hardness in the far TMAZ/HAZ, but could not associate the drop to any microstructural changes. Hence, despite these different studies, the presence of a hardness drop and the associated microstructural transformations in the HAZ remained undecided. An obvious challenge in clarifying the occurrence of a lower hardness in the HAZ has been the significant hardness fluctuation across the PM (as indicated in Table 4)—due to the varying microstructures and phase constituents—that has often masked and convoluted the interpretation of any hardness differences. This, of course, has been compounded by the multiple transformations of the PM phase constituents that transpire over a limited/narrow TMAZ/HAZ. In the present study, the use of 3D hardness maps allowed clear visualization of the hardness drop in both HAZs, even with the hardness fluctuations in Ti-64 and Ti-6242 PMs, and the BSE SEM images effectively differentiated the vanadium-enriched metastable β phase regions. Additionally, it was demonstrated that SRA effectively recovers the hardness in the HAZ due to reverse transformation of the metastable β phase to more equilibrium levels of α and β phases in the microstructure. It is worth mentioning that the impact of this soft metastable/retained β phase in the TMAZ/HAZ of AWed dissimilar alloy joints is thought to be less adverse than the coarsening of grains [62] and/or grain boundary α [80], which are difficult to reverse/refine in titanium alloys. In comparison, reverse transformation of the metastable/retained β phase during SRA enables complete recovery of the hardness, and, in practice, as SRA is integral to the LFW process for mitigating the high tensile and compressive residual stresses in the welds [19], there are no extra post-processing operations needed.

## 5. Tensile Mechanical Properties

**Figure 12:**
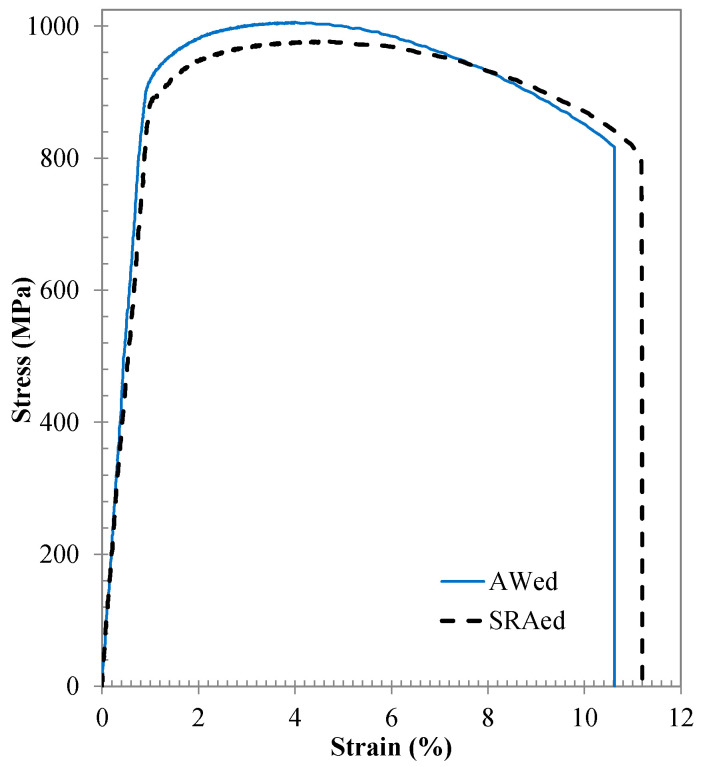
Average stress–strain behavior for dissimilar titanium alloy welds in the AWed and SRAed conditions.

Representative engineering stress–strain curves, generated from static tensile testing of the AWed and SRAed dissimilar joints between Ti-64 and Ti-6242, are shown in Figure 12. The average room temperature tensile properties of the AWed and SRAed dissimilar joints are listed in Table 5, alongside the minimum values for the YS, UTS and El specified in AMS 4911 for Ti-64 and AMS 4919 for Ti-6242. Overall, the mechanical performance of the dissimilar joints between Ti-64 and Ti-6242 in both the AWed and SRAed conditions exceeded the specifications for the Ti-64 and Ti-6242 alloys and failure occurred exclusively in the Ti-64 PM, roughly 5 ± 1 mm away from the CWZ, indicating that the PDZ was stronger, in comparison. In this regard, the joint efficiency (based on the strength of the weld relative to the Ti-64 or Ti-6242 alloys) of the dissimilar titanium alloy welds was 100% in the AWed and SRAed conditions. This finding agrees reasonably well with previous observations and reported tensile properties of similar and dissimilar titanium alloy linear friction welds, as compared in Table 6. In addition, relative to friction stir welds [81] between Ti-64 and Ti-6242 that were reported to have a YS of 790–840 MPa, UTS of 870–900 MPa and El of 1–14%, the linear friction welds in the present study exhibit superior performance with a combination of high strength and good ductility.

The effect of SRA on the AWed properties was a decrease in the YS of 8% (from 935 ± 13 to 862 ± 18 MPa) and UTS of 4% (from 1008 ± 4 to 967 ± 3 MPa), with a slight increase in the El of 1% (from 11 ± 0.1 to 12 ± 0.4). Interestingly, SRA that increased the hardness of the Ti-64 PM to slightly above that of the Ti-6242 PM, did not seem to alter the tensile failure location. This may be an effect of grain structure orientation (rolling versus transverse direction) and/or the difference in microstructure (bimodal versus predominantly primary α). Previously, in the study by Stinville et al. [17] on LFW of dissimilarly textured Ti-64, despite the higher hardness of the workpiece in the rolling direction (355 HV0.5) relative to the transverse direction (315 HV0.5), failure occurred in the former. Moreover, in the present study, the high fraction of interconnected primary α in the Ti-64 microstructure would allow a longer effective slip length that would lower strength relative to the bimodal structure of Ti-6242. This finding corroborates with reported research studies on titanium alloys that have recognized the role of similarly oriented primary α grains on increasing the slip length and contributing as microstructural weak links that lower tensile and fatigue strength [85,86,87].

**Figure 13:**
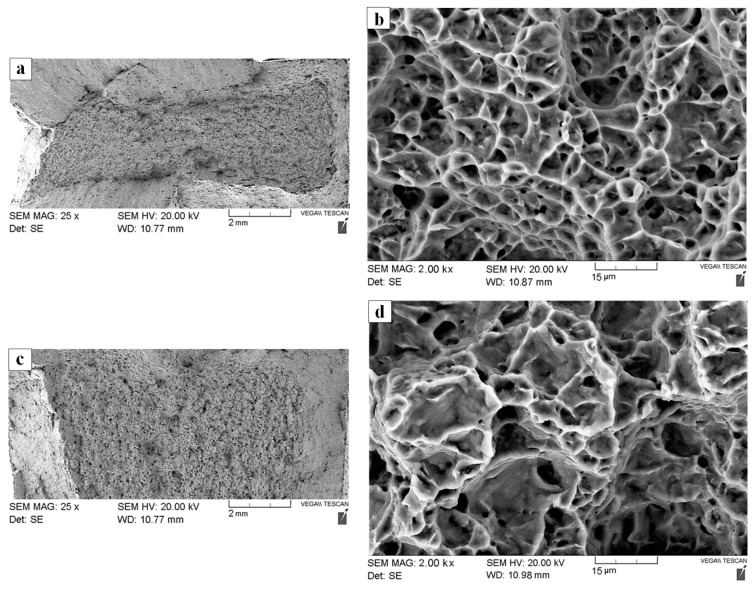
Tensile fracture surface SEM images (**a**) AWed overview; (**b**) AWed magnified; (**c**) SRAed overview; (**d**) SRAed magnified.

After static tensile loading of the dissimilar joints, the fracture surfaces of select specimens were examined using SEM. Overall, there was no noticeable difference in the fracture surface characteristics of the AWed and SRAed specimens at low and high magnifications. Since the AWed and SRAed joints failed exclusively in the Ti-64 PM, fracture surface analysis showed characteristic features of failure in Ti-64 with the observation of ductile ridges and tearing at low magnification (Figure 13a,c). High magnification images of the fracture surfaces revealed a dimpled structure (Figure 13b,d), giving evidence of failure by micro-void formation, followed by growth and coalescence of these micro-pores to form micro-cracks. Concentration of the applied load/stress at the tips of micro-cracks then resulted in crack propagation and final fracture of the specimen in the Ti-64 PM. It is noteworthy that the tensile fracture surfaces were free of large porosities, oxides, or any other inclusions, which are possible for titanium fusion welds that fail due to solidification defects in the weld fusion zone [88]. Previously for dissimilar joints between Ti-64 and Ti-6242 produced by friction stir welding, tensile failure occurred predominantly in the weld and, depending on the processing conditions, both ductile shear and brittle failure in the welds were reported by Kulkarni and Ramulu [81].

## 6. Conclusions

In the present study, the feasibility of joining dissimilar alloys Ti-6Al-4V (Ti-64) and Ti-6Al-2Sn-4Zr-2Mo-0.1Si (Ti-6242) using the linear friction welding (LFW) process was assessed. The following conclusions can be drawn based on the observations of the microstructural characteristics, microhardness evolution and tensile mechanical properties of the dissimilar titanium alloy joints in the as-welded (AWed) and stress relief annealed (SRAed) conditions:

1. The linear friction weld between the Ti-64 and Ti-6242 alloys exhibited intimate bonding without the presence of discontinuities such as pores, voids, or cracks. The flash layer consisted of expelled interface material from both alloys that extruded plastically as a single layer. A distinct wavy weld line remained at the initial weld interface between the Ti-64 and Ti-6242 workpieces, indicating that long-range ordering on diffusion was insignificant across the interface due to the short time at super-transus temperatures during LFW that limited element diffusion and partitioning.
2. Examination of the microstructural evolution across the AWed joint indicated several distinct regions in both the Ti-64 and Ti-6242 sides, including a central weld zone (CWZ), thermomechanically affected zone (TMAZ) and heat-affected zone (HAZ). In the CWZ, the α phase in the room temperature microstructures of the Ti-6242 and Ti-64 alloys completely transformed to the β phase that dynamically recrystallized due to the significant plastic deformation at super-transus temperatures. On cooling after LFW, the CWZ microstructure of refined transformed prior-β grains consisted of martensite (α’). The TMAZ microstructure was heavily deformed, but not recrystallized, which points to sub-transus temperatures in this region. The phase constituents in the TMAZ consisted of elongated and fragmented primary α and transformed prior-β grains with acicular α and retained metastable β phases. Affected only by heat from the LFW process, the HAZ microstructure bore similarity to the adjacent Ti-64 and Ti-6242 parent materials (PMs) but the presence of retained metastable β was identified using backscattered electron imaging.
3. After SRA, the microstructures across the joint indicated tempering of α’ in the CWZ and TMAZ, and transformation of the retained metastable β in the TMAZ and HAZ to equilibrated fractions of α and β phases.
4. 3D microhardness maps across the dissimilar alloy joints in the AWed condition indicated peak values in the CWZ and softening in the HAZ that were related to the respective microstructures, namely refined prior β grains consisting of α’ and PM microstructures with retained metastable β. The TMAZ hardness was comparatively lower than the CWZ due to the lower kinetics for dynamic recrystallization (limited plastic deformation, sub-transus temperatures and partial phase transformations). After SRA, the CWZ hardness decreased by 8% (due to tempering of α’) and the HAZ softening recovered (from the transformation of retained metastable β) without any noticeable change in the TMAZ hardness.
5. The tensile mechanical properties of the dissimilar joints in the AWed and SRAed conditions surpassed the AMS specifications for Ti-64 and Ti-6242. Tensile failure occurred exclusively in the Ti-64 PM through ductile fracture mechanisms.

## Figures and Tables

**Table 1:** Chemical composition of the Ti-6Al-4V (Ti-64) and Ti-6Al-2Sn-4Zr-2Mo-0.1Si (Ti-6242) parent materials (PMs) (wt.%).

Element	Ti-64	Ti-6242
Al	6.08	6.12
V	4.11	-
Sn	-	2.18
Zr	-	4.35
Mo	-	2.19
Si	-	0.1
Fe	0.2	0.1
C	0.02	0.01
H	0.003	0.009
O	0.09	0.10
N	0.02	0.01
Ti	Balance	Balance

**Table 2:** Typical properties of Ti-64 and Ti-6242 PMs [40,41].

Properties	Ti-64	Ti-6242
β transus (°C)	980	990
Density (g·cm^3^)	4.43	4.54
Hardness (HV)	330	333
Ultimate Tensile Strength (MPa)	970	1000
Yield Strength (MPa)	900	930
Elongation (%)	14	15
Elastic Modulus (GPa)	113.8	118.0

**Table 3:** Process parameters used for linear friction welding (LFW) of Ti-64 to Ti-6242.

Frequency (Hz)	Amplitude (mm)	Pressure (MPa)	Shortening (mm)
50	2	90	2

**Table 4:** Comparison of hardness in CWZ relative to PM.

Joint Type	Reference	State	Microhardness (HV)		Difference
			PM	CWZ	(%)
Dissimilar Ti-64 to Ti-6242	Present Study	AWed	326 ± 4 (Ti-64) 345 ± 8 (Ti-6242)	398 ± 3	22
Dissimilar Ti-64 to Ti-6242	Present Study	SRAed	352 ± 5 (Ti-64) 342 ± 9 (Ti-6242)	367 ± 2	7
Similar Ti-64	Sun et al. [14]	AWed	330	390	18
	Yesid and Londono [78]		335 ± 5	405 ± 1	21
	Guo et al. [31]		300–330	360–370	23
	Kuroki et al. [77]		325–328	375–400	23
	Wanjara and Jahazi [39]		321–349	398 ± 3	24
	Stinville et al. [17]		315–355	395	25
	Romero et al. [16]		328 ± 20	425 ± 10	30
	Li et al. [15]		302 ± 20	422 ± 11	40
Similar Ti-6242	García and Morgeneyer [69]	AWed	330	420	27
	Ballat-Durand et al. [74]		340	475	40

**Table 5:** Average mechanical properties of dissimilar joints between Ti-64 and Ti-6242.

Material	Reference	State	YS (MPa)	UTS (MPa)	El (%)	Failure
Dissimilar Ti-64 to Ti-6242	Present Study	AWed	935 ± 13	1008 ± 4	11 ± 0.1	Ti-64 PM
		SRAed	862 ± 18	967 ± 3	12 ± 0.4	Ti-64 PM
Ti-64 PM (25 mm thick)	AMS 4911P [82]	Mill Annealed	827	893	10	NA
Ti-6242 PM (25 mm thick)	AMS 4919J [83]	Duplex Annealed	827	896	10	NA

**Table 6:** Comparison of the tensile properties of similar and dissimilar titanium alloy linear friction welds.

Joint Type	Reference	State	YS (MPa)	UTS (MPa)	El (%)	Failure
Dissimilar Ti-64 to Ti-6242	Present Study	AWed	935 ± 13	1008 ± 4	11 ± 0.1	Ti-64 PM
		SRAed	862 ± 18	967 ± 3	12 ± 0.4	Ti-64 PM
Dissimilar Ti-64 to Ti-6246	Corzo et al. [30]	AWed	986	1032	14	Ti-64 PM
Dissimilar Ti-6242 to Ti-6246	Corzo et al. [30]	AWed	1026	1093	12	Ti-6242 PM
Similar Ti-64	Wanjara and Jahazi [39]	Awed *	991–999	1031–1054	11.2–12.2	PM
	Li et al. [15]	AWed	795–938	876–998	7.1–13.3	PM
	Filipo et al. [84]	AWed	957	1015	14.5	PM
Similar Ti-6242	Garcia et al. [69]	AWed	875	960	9.2	Oscillating PM

* Excluding welding conditions producing defects (oxides, pores) in joint.
